# Development and usability testing of a patient digital twin for critical care education: a mixed methods study

**DOI:** 10.3389/fmed.2023.1336897

**Published:** 2024-01-11

**Authors:** Lucrezia Rovati, Phillip J. Gary, Edin Cubro, Yue Dong, Oguz Kilickaya, Phillip J. Schulte, Xiang Zhong, Malin Wörster, Diana J. Kelm, Ognjen Gajic, Alexander S. Niven, Amos Lal

**Affiliations:** ^1^Department of Medicine, Division of Pulmonary and Critical Care Medicine, Mayo Clinic, Rochester, MN, United States; ^2^School of Medicine and Surgery, University of Milano-Bicocca, Milan, Italy; ^3^Department of Information Technology, Mayo Clinic, Rochester, MN, United States; ^4^Department of Anesthesiology and Perioperative Medicine, Mayo Clinic, Rochester, MN, United States; ^5^Department of Quantitative Health Sciences, Division of Clinical Trials and Biostatistics, Mayo Clinic, Rochester, MN, United States; ^6^Department of Industrial and Systems Engineering, University of Florida, Gainesville, FL, United States; ^7^Center for Anesthesiology and Intensive Care Medicine, Department of Anesthesiology, University Medical Center Hamburg-Eppendorf, Hamburg, Germany

**Keywords:** critical care, medical education, patient-specific modeling, simulation training, patient safety, medical intensive care unit

## Abstract

**Background:**

Digital twins are computerized patient replicas that allow clinical interventions testing *in silico* to minimize preventable patient harm. Our group has developed a novel application software utilizing a digital twin patient model based on electronic health record (EHR) variables to simulate clinical trajectories during the initial 6 h of critical illness. This study aimed to assess the usability, workload, and acceptance of the digital twin application as an educational tool in critical care.

**Methods:**

A mixed methods study was conducted during seven user testing sessions of the digital twin application with thirty-five first-year internal medicine residents. Qualitative data were collected using a think-aloud and semi-structured interview format, while quantitative measurements included the System Usability Scale (SUS), NASA Task Load Index (NASA-TLX), and a short survey.

**Results:**

Median SUS scores and NASA-TLX were 70 (IQR 62.5–82.5) and 29.2 (IQR 22.5–34.2), consistent with good software usability and low to moderate workload, respectively. Residents expressed interest in using the digital twin application for ICU rotations and identified five themes for software improvement: clinical fidelity, interface organization, learning experience, serious gaming, and implementation strategies.

**Conclusion:**

A digital twin application based on EHR clinical variables showed good usability and high acceptance for critical care education.

## 1 Introduction

Medical errors remain a major cause of morbidity, mortality, and cost in the US healthcare system (1). The intensive care unit (ICU) is particularly prone to preventable adverse events due to the complexity of care delivery and the patient severity of illnesses (2). The fast pace and high acuity of critical care practice can also limit opportunities for trainee autonomy. Providing a safe environment to practice decision-making in this setting may improve the ICU educational experience, care processes, and patient-centered outcomes (3).

Digital twins are virtual models that simulate the behavior of real objects in a digital environment. With the increasing availability of electronic health record (EHR) and sensor-derived patient data, digital twins hold significant potential applications within the healthcare sector (4, 5). In particular, digital twin technology enables the creation of computerized patient replicas, simulating diverse clinical scenarios and intervention testing *in silico* to reduce avoidable risk in real patients (6).

Digital twins offer particular promise in critical care, where large quantities of data are continuously available, and the risk to patient safety posed by medical interventions is often significant (7, 8). The benefits of a digital twin patient model to inform clinical decision-making in critical illness have been previously proposed (9–12). Digital twins could also be adapted for critical care education, allowing learners to simulate the effects of various interventions and explore their potential outcomes in a controlled, virtual environment without negative patient impacts (13, 14). Compared to conventional virtual patient simulation models, digital twins provide users with a more authentic experience in complex illness management by incorporating real-time, EHR-derived patient data into comprehensive computational models (15–17).

Our group has previously described the design and validation of a novel digital twin based on EHR clinical variables to model critically ill patients with sepsis for bedside decision support (11, 13). In this model, major organ systems interact based on programmed expert rules to recreate and predict the future patient state in response to specific clinical interventions. In this work, we developed a novel application software utilizing this digital twin patient model to simulate clinical trajectories during the initial 6 h of critical illness. This study aimed to assess the usability, workload, and acceptance of the digital twin application software for critical care education in a cohort of internal medicine residents.

## 2 Materials and methods

**Figure 1:**
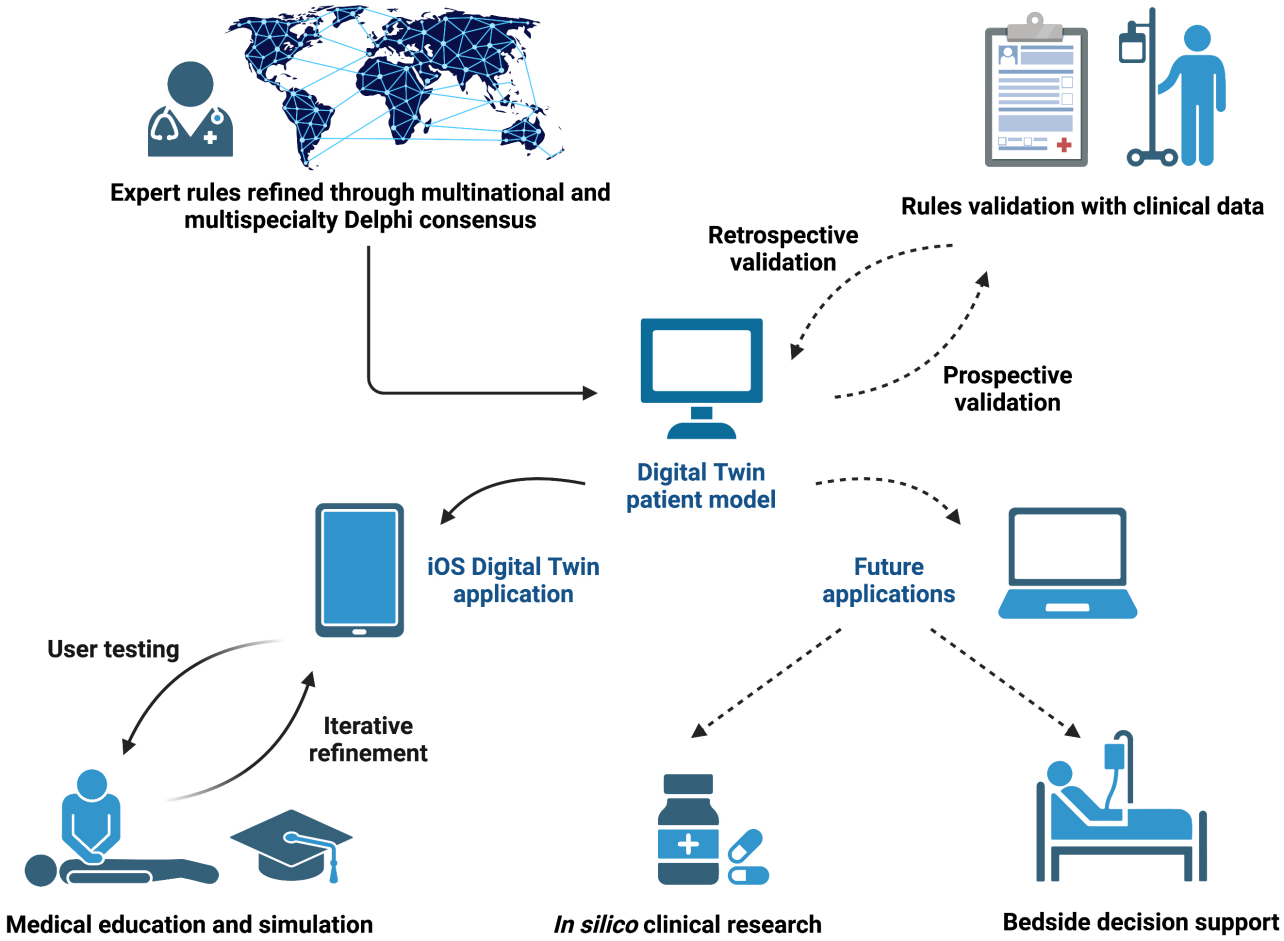
Overview of the critical care patient digital twin project. The digital twin patient model was designed based on expert rules and electronic health record clinical variables. In this study, we focused on the development and usability testing of an iOS digital twin application for critical care education (solid arrows). After further prospective and retrospective validation with clinical data, future applications of the digital twin model include *in silico* clinical trials and bedside decision support (dashed arrows). This figure was created with BioRender.com.

Figure 1 provides an overview of the overall critical care patient digital twin project.

This study comprised three sequential phases:

1. Design and coding of a digital twin patient model based on EHR clinical variables and expert rules to simulate patient trajectories during the initial 6 h of critical illness.
2. Development of the user interface for an iOS digital twin application software designed for critical care education delivery.
3. Usability testing of the digital twin application software with a cohort of internal medicine residents and collection of user feedback for iterative software improvement.

### 2.1 Digital twin patient model design and coding

The digital twin patient model tested in this study focused on physiologic interactions and medication effects relevant to the initial 6 h, or golden hours, of critical illness (18). Variables included in the model comprised clinical data commonly displayed in the ICU EHR. Expert rules describing the interactions between the seven major organ systems (neurologic, respiratory, cardiovascular, gastrointestinal, renal, immunologic, and hematologic) were developed using available literature and current clinical practice guidelines and refined using a modified Delphi panel of international critical care experts (11, 13, 19, 20). Medication effects and pharmacokinetic rules were derived from publicly available drug databases. The model was based on 70 total expert rules and iteratively improved based on feedback from the investigator group. A detailed description of model design and coding, together with two examples of expert rules, are presented in the Supplementary Materials and Methods. The rules that describe the physiologic interactions between the organ system variables are represented graphically in Supplementary Figure 1.

### 2.2 Digital twin application software development

The digital twin pilot application software tested in this study was developed on iOS using Swift programming language and Xcode integrated development environment version 14.2. User testing sessions were performed with a tablet version of the iOS digital twin application.

**Figure 2:**
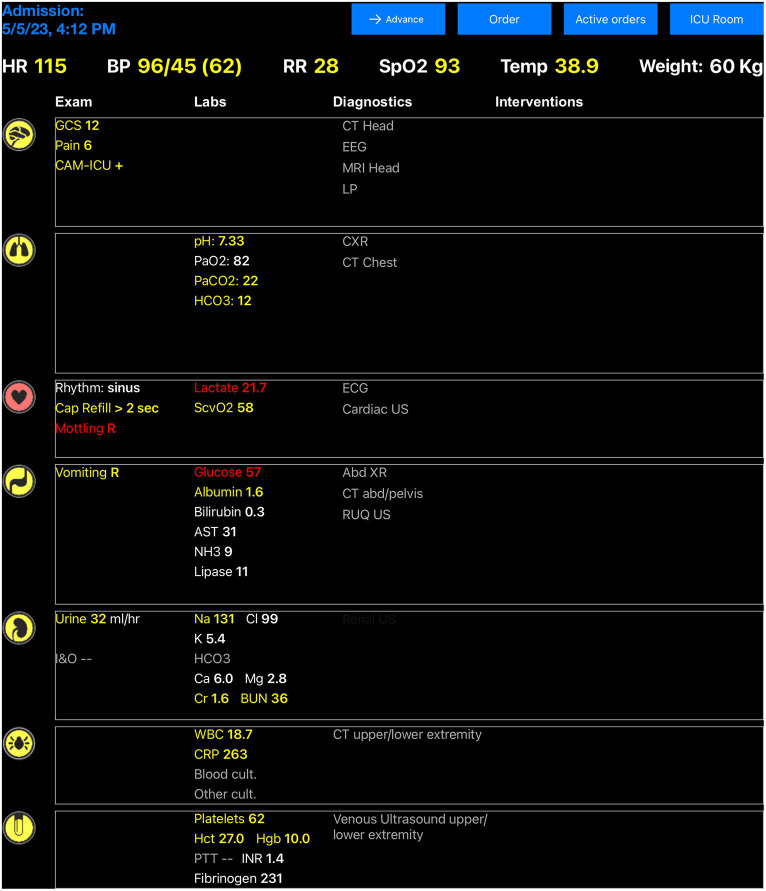
Electronic health record interface of the digital twin application software. Clinical variables included in the digital twin patient model are represented in the electronic health record screen and updated based on expert rules triggered by clinical interventions or changes in the patient’s clinical status. White color indicates that a clinical variable is in its normal range and no intervention is needed, while yellow or red colors indicate a variable disturbance that would require urgent or emergent action.

The user interface of the digital twin application software consists of a case selection screen, a patient room screen, an EHR screen, and an order entry screen. Users can select a case from a list of virtual clinical scenarios that include urosepsis, chronic obstructive pulmonary disease exacerbation, acute respiratory failure due to pneumonia, acute liver failure, gastrointestinal bleeding, myocardial infarction, and acute decompensated congestive heart failure. Each clinical scenario incorporates specific organ system variable alterations into the initial virtual patient presentation. The user can review the patient’s history and physical examination findings on the patient room screen. The EHR screen displays the most relevant data for critical care decision-making, organized by organ systems and color-coded based on the degree of abnormality (21). These data are divided into physical examination, laboratory testing, and other diagnostic findings. Clinical interventions performed by the user are displayed on the EHR screen, maintaining the organ system organization (Figure 2).

After using the order entry screen to initiate a diagnostic test or intervention, the user can advance the timeline (by 15-min intervals for the first hour, then by one-hour intervals until the 6-h endpoint of the simulation) to trigger the associated expert rules coded in the digital twin patient model. The expert rule engine determines which rules are executed based on the interventions ordered and the current value of each organ system variable, which defines the patient’s clinical status. The effects of these rules are displayed as changes in the relevant clinical variables presented in the EHR, which reflect the patient’s physiological response to the different interventions.

### 2.3 Usability testing of the digital twin application software

#### 2.3.1 Study design and setting

To explore the usability of the digital twin application software as an educational tool in critical care, we collected both quantitative and qualitative data during seven user testing sessions with internal medicine resident volunteers performed at the Mayo Clinic, Rochester, from August 2022 to June 2023. Participants were compensated for their time with a gift card. The study protocol was evaluated and approved as exempt by the Mayo Clinic Institutional Review Board (IRB 21-010982; study title “Critical Care Coaching with an Electronic Health Record Digital Twin”; approval date 11/8/2021) after review by the Mayo Clinic Education Research Committee and the Mayo Clinic Internal Medicine Research in Education Group. The study was conducted in accordance with the ethical standards of the responsible institutional committee on human experimentation and with the Helsinki Declaration of 1975, as most recently amended. Verbal consent was obtained from the participants before each testing session.

#### 2.3.2 Qualitative data collection and analysis

During user testing sessions, residents interacted for 15 min with a simulated case, describing their experience using a think-aloud and semi-structured interview format. The urosepsis case was used for all the user testing sessions to ensure consistency. Each case scenario and debriefing session was recorded, de-identified, transcribed, and analyzed for common themes. Qualitative data were used to refine the software and identify possible digital twin application implementation strategies in the current critical care curriculum.

#### 2.3.3 Quantitative data collection and analysis

The System Usability Scale (SUS), NASA Task Load Index (NASA-TLX), and two survey questions were administered to each user at the end of the simulation session to collect quantitative information on software usability, workload, and learner acceptance. SUS is a measure of usability consisting of 10 questions with five options each (22). The final score ranges from 0 (low usability) to 100 (high usability). NASA-TLX measures perceived workload and evaluates six domains: mental demand, physical demand, temporal demand, performance, effort, and frustration (23). Each domain is scored from 0 (low workload) to 100 (high workload) in 5-point steps, then the unweighted average of the subscale scores is obtained. The survey questions explored how residents would consider using the digital twin application to prepare for or as part of their medical ICU rotation. De-identified data were collected and managed using Research Electronic Data Capture version 8.11.11 (REDCap, Vanderbilt University, Nashville, Tennessee, USA). Statistical analysis was performed using GraphPad Prism version 9.0.0 (GraphPad Software, San Diego, California, USA). To summarize the results, median (interquartile range, IQR) and counts (%) were used.

## 3 Results

Thirty-five post-graduate year one internal medicine residents participated in the user testing sessions of the digital twin application software. All residents were recruited during pre-planned central venous catheter procedural workshops conducted before the start of their medical ICU rotation.

### 3.1 Digital twin application software usability, workload, and acceptance

**Figure 3:**
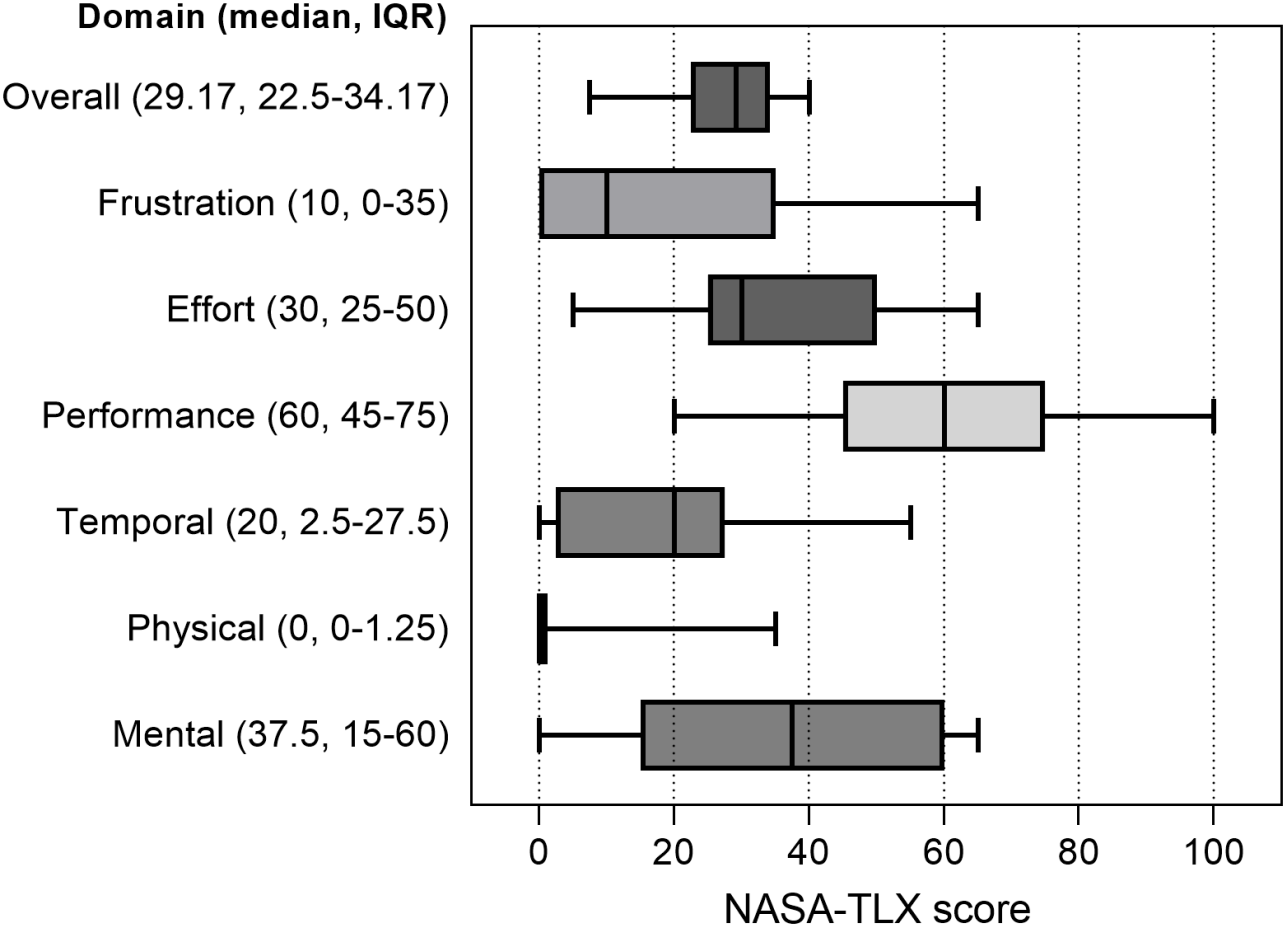
Perceived workload of the digital twin application software as measured by the NASA Task Load Index. Overall and single-domain NASA Task Load Index (NASA-TLX) scores were obtained for each resident during user testing sessions (*n* = 35). Box plots represent median values (solid bar), interquartile range (IQR, margins of the box), and minimum and maximum values (whiskers).

The average SUS score in our cohort was 70 (IQR 62.5–82.5), consistent with good software usability (22). The average NASA-TLX score was 29.2 (IQR 22.5–34.2), reflecting a low to moderate workload (24). The scores of each NASA-TLX domain are presented in Figure 3. The greatest perceived difficulty was the successful performance of required tasks, while physical and temporal demand and frustration levels were considered low. Mental demand and overall effort were rated as moderately high. More than 60% of residents indicated that they would use the digital twin application for a moderate amount or a great deal of time to prepare for and as part of their medical ICU rotation (Table 1).

**Table 1:** Results from the survey questions assessing the willingness of residents to use the digital twin application for medical ICU orientation and education.

Responses (*n* = 35)	Would you use this tool to prepare for medical ICU rotation?	Would you use this tool as part of your medical ICU rotation?
Never	0 (0%)	0 (0%)
Rarely	2 (6%)	5 (14%)
Occasionally	11 (31%)	6 (17%)
A moderate amount	15 (43%)	17 (49%)
A great deal	7 (20%)	7 (20%)

### 3.2 User feedback for iterative software improvement

Resident comments for iterative software improvement were clustered in five domains, summarized in Table 2. Learners highlighted the importance of the digital twin application delivering a realistic clinical experience, including interactions with the virtual patient and simulated clinical environment and a plausible timeline for scenario progression. Residents also suggested that the EHR interface of the application software should be similar to the commercial product they use in the clinical environment. This would help them to learn to gather and interpret results and navigate the ordering process efficiently. They felt the digital twin application was most helpful in learning medication dosing and effects, enhancing pattern recognition, and improving their understanding of current guidelines through practice managing common ICU scenarios. Learners were mainly interested in a serious gaming experience to test their clinical skills in a safe environment, with a final evaluation reflected by a performance score attributed at the end of each scenario. Residents expressed a willingness to utilize the digital twin application before and during medical ICU rotations; however, they highlighted that their busy clinical schedules pose a significant obstacle to the implementation of the application, as they have limited free time available to use it. To address this issue, the internal medicine residents proposed incorporating practice sessions utilizing the digital twin application software into the current critical care education curriculum.

**Table 2:** Main themes identified during user testing sessions.

Theme	Sub-themes
Clinical fidelity	Interaction with the virtual patient
	Interaction with the virtual environment
	Virtual time progression similar to real life
Interface organization	Avoid information overload
	Reflect on what is used in daily clinical practice
Learning experience	Learn and practice using medications, including dosing and effects, in common ICU scenarios
	Blend simulation with formal explanations
	Accurate, up-to-date information reflecting current guidelines
Serious gaming	Test clinical skills in a safe environment
	Obtain a performance score at the end of the simulation
Implementation barriers and strategies	Limited free time to use the application software
	Integration of practice sessions with the digital twin application into the existing critical care education curriculum

## 4 Discussion

This study presents the development and usability testing of a novel application software for critical care education built upon a digital twin patient model based on EHR clinical variables. The digital twin application allows physicians-in-training to test clinical interventions on virtual patients, fostering autonomy and advancing clinical skills in a safe environment that does not expose real patients to preventable harm. Digital twin application testing in a cohort of internal medicine residents suggests high software usability and learner willingness to use this tool to enhance their medical ICU rotation experience.

Although simulation-based education can improve learner confidence and knowledge, evidence supporting superior learning outcomes over more traditional educational delivery methods has varied based on the learning goals (25–28). One notable advantage of simulation is its capacity to offer standardized, reproducible clinical scenarios within a risk-free learning environment, with clear patient safety benefits (29, 30). Emerging technologies, including medical simulation mobile applications and virtual reality, provide further opportunities for remote and on-demand training using simulated clinical cases, providing a consistent framework of residency training experiences that is more cost-effective than traditional high-fidelity simulation (31–33). In addition to providing flexible, efficient online opportunities for deliberate practice, digital twin technology can also integrate real-time patient data to create highly accurate and realistic virtual patient models (4). Indeed, residents underlined the importance of clinical fidelity during user testing sessions of our digital twin application software, including appropriate and realistic responses to clinical interventions. The major disadvantage of the digital twin and other virtual simulation applications is that they do not allow for hands-on practice of the clinical interventions being tested, for which traditional manikin-based simulation remains the gold standard.

Residents acknowledged the potential of the digital twin application to enhance their critical care educational experience. However, they identified clinical schedule demands as the primary obstacle to effectively implementing this tool. In addition to dedicating time within the current critical care curriculum to practice using the digital twin application, residents suggested incorporating additional gamification features, such as a point and badge system, to increase user engagement. Serious gaming has been utilized in various medical education settings, including critical care and emergency medicine, and has been shown to improve knowledge retention and clinical competence (34, 35). However, most studies to date have lacked well-defined control groups, and further research is needed to better understand the benefits of this educational delivery method on learning outcomes, together with the most appropriate learner group, educational context, and experience to achieve these goals (36, 37).

Clinical data display was an important theme raised during software development and user testing sessions. Residents must rapidly learn to identify and review a significant volume of data associated with each patient in the ICU setting. Reviewing this clinical information takes significant time, and this task can feel overwhelming for new trainees without an organized approach (21, 38, 39). To address these challenges, the digital twin application interface displays only the most relevant data for treating critical illness. These data are also organized by organ system and color-coded based on the degree of physiological disturbance and need for action (Figure 2). This user interface design has been shown to reduce time to clinical task completion, task load, and errors of cognition in the ICU when compared with standard EHR interfaces (40, 41). During user testing sessions, residents acknowledged the potential usefulness of the system-based interface organization in the ICU context. However, they also emphasized the differences between this data display and the interface they regularly encounter in their clinical duties. They specifically highlighted the importance of practicing navigation within standard EHR systems at the beginning of their training. This situation creates a dilemma between two distinct learning objectives: the need for clear data presentation to minimize cognitive load and support deliberate practice in critical care decision-making versus data presentation that closely resembles the clinical EHR interface to enhance order entry efficiency through practice but potentially hinders the development of clinical reasoning in typical critical care scenarios. The challenges of adapting to the new interface might also have contributed to the moderately high NASA-TLX scores recorded in the domains of mental demand, successful task performance, and overall effort recorded during testing sessions. Additionally, the significant variations observed in the performance, mental demand, effort, and frustration domains of the score could indicate differences among residents in terms of their critical care knowledge and problem-solving capabilities rather than being attributed solely to the interface itself (42). This subject will require more targeted studies to qualify further.

The digital twin application software offers a convenient, low-cost alternative to enhance the current delivery of critical care education to learners at various levels of experience. This is the first time that digital twin technology has been applied to critical care education. The major strength of our digital twin patient model resides in using transparent pathophysiological relationships to derive expert rules, which have been refined using multinational and multi-specialty Delphi consensus (11, 19). Digital twins can also be developed as purely data-driven models that do not consider causal pathways of diseases, but the lack of clarity in how these physiologic responses are derived creates significant barriers to their acceptance by bedside clinicians (43, 44). To provide clinicians with a better understanding of how the underlying model reaches its output state, future iterations of the digital twin application will offer visualization of pathophysiological relationships using directed acyclic graphs in the user interface (45, 46). The purpose of this methodology for digital twin model design and the user-centered software development process described in this work is to facilitate technology adoption and address the cognitive, emotional, and contextual concerns of clinicians who will utilize this tool (47–49). In the future, the digital twin model will be connected to the current EHR system, allowing continuous update based on real-time patient data to support clinical decision-making, clinical research, and medical education (Figure 1). This will allow clinicians at all experience levels to practice decision-making skills in a safe environment using actual, real-time cases encountered during daily ICU practice. When this step is accomplished, important ethical and regulatory issues must be considered before implementing this novel tool in daily clinical practice (44, 50).

This study has some limitations. First, the digital twin patient model described in this work has been tested on simulated clinical scenarios and on a relatively small cohort of patients with sepsis (11). We plan to prospectively validate this model on a larger cohort of critically ill patients importing real-time EHR data into the application software and further refine expert rules based on these and additional retrospective data. Second, only a limited number of users at a single center participated in the usability testing of the digital twin application software. In addition, all users belonged to a cohort of internal medicine residents with no previous ICU experience, which limit the generalizability of the results. We plan to continue the user testing sessions to iteratively improve the current digital twin application software, involving more senior residents, fellows, and staff intensivists with different experience levels to systematically validate this educational tool’s performance and learning outcomes and compare it to more conventional educational techniques.

## 5 Conclusion

Our novel digital twin application software based on EHR clinical variables proved highly usable and well accepted by first-year internal medicine residents, and their feedback will inform further iterative improvement of its interface. The digital twin application software provides an attractive, realistic, low-cost option to teach critical care clinical decision-making. It offers opportunities for deliberate practice in a virtual environment, building experience and confidence on real-time ICU cases, which may result in greater opportunities for graduated learner autonomy at the bedside and reduced risk of medical errors.

## Data availability statement

The raw data supporting the conclusions of this article will be made available by the authors, without undue reservation.

## Ethics statement

The studies involving humans were approved by Mayo Clinic Institutional Review Board. The studies were conducted in accordance with the local legislation and institutional requirements. The participants provided their written informed consent to participate in this study.

## Author contributions

LR: Conceptualization, Data curation, Formal analysis, Investigation, Methodology, Visualization, Writing – original draft. PG: Conceptualization, Investigation, Methodology, Writing – original draft. EC: Conceptualization, Software, Writing – original draft. YD: Conceptualization, Investigation, Project administration, Supervision, Writing – review & editing. OK: Conceptualization, Investigation, Writing – review & editing. PS: Conceptualization, Funding acquisition, Methodology, Supervision, Writing – review & editing. XZ: Conceptualization, Funding acquisition, Methodology, Software, Supervision, Writing – review & editing. MW: Conceptualization, Writing – review & editing. DK: Conceptualization, Supervision, Writing – review & editing. OG: Conceptualization, Methodology, Supervision, Writing – review & editing. AN: Conceptualization, Funding acquisition, Methodology, Supervision, Writing – review & editing. AL: Conceptualization, Funding acquisition, Methodology, Supervision, Writing – review & editing.

## References

[1] Anderson JG Abrahamson K. Your health care may kill you: medical errors. Stud Health Technol Inform. (2017) 234:13–7. doi: 10.3233/978-1-61499-742-9-13 PMID: 28186008

[2] Ahmed AH Giri J Kashyap R Singh B Dong Y Kilickaya O. Outcome of adverse events and medical errors in the intensive care unit: a systematic review and meta-analysis. Am J Med Qual. (2015) 30:23–30. doi: 10.1177/1062860613514770 24357344

[3] Herzog TL Sawatsky AP Kelm DJ Nelson DR Park JG Niven AS. The resident learning journey in the medical intensive care unit. ATS Scholar. (2022) 4:177–90. doi: 10.34197/ats-scholar.2022-0103OC PMC10391714 37533538

[4] Sun T He X Song X Shu L Li Z. The digital twin in medicine: a key to the future of healthcare? Front Med. (2022) 9:907066. doi: 10.3389/fmed.2022.907066, PMID: 35911407 PMC9330225

[5] Sun T He X Li Z. Digital twin in healthcare: recent updates and challenges. Digit Health. (2023) 9:205520762211496. doi: 10.1177/20552076221149651 PMC9830576 36636729

[6] Sahal R Alsamhi SH Brown KN. Personal digital twin: a close look into the present and a step towards the future of personalised healthcare industry. Sensors. (2022) 22:5918. doi: 10.3390/s22155918, PMID: 35957477 PMC9371419

[7] Chase JG Preiser JC Dickson JL Pironet A Chiew YS Pretty CG. Next-generation, personalised, model-based critical care medicine: a state-of-the art review of in silico virtual patient models, methods, and cohorts, and how to validation them. Biomed Eng Online. (2018) 17:24. doi: 10.1186/s12938-018-0455-y, PMID: 29463246 PMC5819676

[8] Komorowski M Celi LA Badawi O Gordon AC Faisal AA. The artificial intelligence clinician learns optimal treatment strategies for sepsis in intensive care. Nat Med. (2018) 24:1716–20. doi: 10.1038/s41591-018-0213-5 30349085

[9] Ang CYS Lee JWW Chiew YS Wang X Tan CP Cove ME. Virtual patient framework for the testing of mechanical ventilation airway pressure and flow settings protocol. Comput Methods Prog Biomed. (2022) 226:107146. doi: 10.1016/j.cmpb.2022.107146, PMID: 36191352

[10] Chakshu NK Nithiarasu P. An AI based digital-twin for prioritising pneumonia patient treatment. Proc Inst Mech Eng H. (2022) 236:1662–74. doi: 10.1177/09544119221123431 36121054 PMC9647318

[11] Lal A Li G Cubro E Chalmers S Li H Herasevich V. Development and verification of a digital twin patient model to predict specific treatment response during the first 24 hours of sepsis. Crit Care Explor. (2020) 2:e0249. doi: 10.1097/CCE.0000000000000249, PMID: 33225302 PMC7671877

[12] Lonsdale H Gray GM Ahumada LM Yates HM Varughese A Rehman MA. The perioperative human digital twin. Anesth Analg. (2022) 134:885–92. doi: 10.1213/ANE.0000000000005916, PMID: 35299215

[13] Trevena W Lal A Zec S Cubro E Zhong X Dong Y. Modeling of critically ill patient pathways to support intensive care delivery. IEEE Robot Automation Lett. (2022) 7:7287–94. doi: 10.1109/LRA.2022.3183253

[14] Zackoff MW Rios M Davis D Boyd S Roque I Anderson I. Immersive virtual reality onboarding using a digital twin for a new clinical space expansion: a novel approach to large-scale training for health care providers. J Pediatr. (2023) 252:7–10.e3. doi: 10.1016/j.jpeds.2022.07.031, PMID: 35973444

[15] Kononowicz AA Woodham LA Edelbring S Stathakarou N Davies D Saxena N. Virtual patient simulations in health professions education: systematic review and meta-analysis by the digital health education collaboration. J Med Internet Res. (2019) 21:e14676. doi: 10.2196/14676, PMID: 31267981 PMC6632099

[16] Eissing T Kuepfer L Becker C Block M Coboeken K Gaub T. A computational systems biology software platform for multiscale modeling and simulation: integrating whole-body physiology, disease biology, and molecular reaction networks. Front Physiol. (2011) 2:4. doi: 10.3389/fphys.2011.00004 21483730 PMC3070480

[17] McDaniel M Keller JM White S Baird A. A whole-body mathematical model of Sepsis progression and treatment designed in the BioGears physiology engine. Front Physiol. (2019) 10:1321. doi: 10.3389/fphys.2019.01321, PMID: 31681022 PMC6813930

[18] Hauffe T Krüger B Bettex D Rudiger A. Shock management for cardio-surgical ICU patients – the golden hours. Card Fail Rev. (2015) 1:75–82. doi: 10.15420/cfr.2015.1.2.75, PMID: 28785436 PMC5490875

[19] Dang J Lal A Montgomery A Flurin L Litell J Gajic O. Developing DELPHI expert consensus rules for a digital twin model of acute stroke care in the neuro critical care unit. BMC Neurol. (2023) 23:161. doi: 10.1186/s12883-023-03192-9, PMID: 37085850 PMC10121414

[20] Montgomery AJ Litell J Dang J Flurin L Gajic O Lal A. Gaining consensus on expert rule statements for acute respiratory failure digital twin patient model in intensive care unit using a Delphi method. Biomol Biomed. (2023) 23:1108–17. doi: 10.17305/bb.2023.9344, PMID: 37431943 PMC10655890

[21] Pickering BW Herasevich V Ahmed A Gajic O. Novel representation of clinical information in the ICU: developing user interfaces which reduce information overload. Appl Clin Inform. (2010) 01:116–31. doi: 10.4338/ACI-2009-12-CR-0027 PMC3632280 23616831

[22] Bangor A Kortum PT Miller JT. An empirical evaluation of the system usability scale. Int J Hum Comput Interact. (2008) 24:574–94. doi: 10.1080/10447310802205776

[23] Hart SG. NASA-task load index (NASA-TLX); 20 years later. Proc Hum Fact Ergon Soc Ann Meet. (2006) 50:904–8. doi: 10.1177/154193120605000909

[24] Grier RA. How high is high? A meta-analysis of NASA-TLX global workload scores. Proc Hum Fact Ergon Soc Ann Meet. (2015) 59:1727–31. doi: 10.1177/1541931215591373

[25] Zendejas B Brydges R Wang AT Cook DA. Patient outcomes in simulation-based medical education: a systematic review. J Gen Intern Med. (2013) 28:1078–89. doi: 10.1007/s11606-012-2264-5, PMID: 23595919 PMC3710391

[26] Cook DA Hatala R Brydges R Zendejas B Szostek JH Wang AT. Technology-enhanced simulation for health professions education: a systematic review and meta-analysis. JAMA. (2011) 306:978–88. doi: 10.1001/jama.2011.1234 21900138

[27] Schroedl CJ Corbridge TC Cohen ER Fakhran SS Schimmel D McGaghie WC. Use of simulation-based education to improve resident learning and patient care in the medical intensive care unit: a randomized trial. J Crit Care. (2012) 27:219.e7–219.e13. doi: 10.1016/j.jcrc.2011.08.006, PMID: 22033049

[28] Steadman RH Coates WC Huang YM Matevosian R Larmon BR McCullough L. Simulation-based training is superior to problem-based learning for the acquisition of critical assessment and management skills. Crit Care Med. (2006) 34:151–7. doi: 10.1097/01.CCM.0000190619.42013.94 16374169

[29] Seam N Lee AJ Vennero M Emlet L. Simulation training in the ICU. Chest. (2019) 156:1223–33. doi: 10.1016/j.chest.2019.07.011, PMID: 31374210 PMC6945651

[30] Hester RL Pruett W Clemmer J Ruckdeschel A. Simulation of integrative physiology for medical education. Morphologie. (2019) 103:187–93. doi: 10.1016/j.morpho.2019.09.004 31563456 PMC7219081

[31] Chandran VP Balakrishnan A Rashid M Pai Kulyadi G Khan S Devi ES. Mobile applications in medical education: a systematic review and meta-analysis. PLoS One. (2022) 17:e0265927. doi: 10.1371/journal.pone.0265927, PMID: 35324994 PMC8947018

[32] Jiang H Vimalesvaran S Wang JK Lim KB Mogali SR Car LT. Virtual reality in medical students’ education: scoping review. JMIR Med Educ. (2022) 8:e34860. doi: 10.2196/34860, PMID: 35107421 PMC8851326

[33] Haerling KA. Cost-utility analysis of virtual and mannequin-based simulation. Simul Healthc. (2018) 13:33–40. doi: 10.1097/SIH.0000000000000280, PMID: 29373382

[34] Dankbaar ME Roozeboom MB Oprins EA Rutten F van Merrienboer JJ van Saase JL. Preparing residents effectively in emergency skills training with a serious game. Simul Healthc. (2017) 12:9–16. doi: 10.1097/SIH.0000000000000194, PMID: 27764018 PMC5291282

[35] Donovan CM Cooper A Kim S. Ready patient one: how to turn an in-person critical care simulation scenario into an online serious game. Cureus. (2021) 13:e17746. doi: 10.7759/cureus.17746, PMID: 34659959 PMC8494055

[36] Gorbanev I Agudelo-Londoño S González RA Cortes A Pomares A Delgadillo V. A systematic review of serious games in medical education: quality of evidence and pedagogical strategy. Med Educ Online. (2018) 23:1438718. doi: 10.1080/10872981.2018.1438718 29457760 PMC5827764

[37] van Gaalen AEJ Brouwer J Schönrock-Adema J Bouwkamp-Timmer T Jaarsma ADC Georgiadis JR. Gamification of health professions education: a systematic review. Adv Health Sci Educ Theory Pract. (2021) 26:683–711. doi: 10.1007/s10459-020-10000-3 33128662 PMC8041684

[38] Pickering BW Litell JM Herasevich V Gajic O. Clinical review: the hospital of the future - building intelligent environments to facilitate safe and effective acute care delivery. Crit Care. (2012) 16:220. doi: 10.1186/cc11142, PMID: 22546172 PMC3681335

[39] Nolan ME Cartin-Ceba R Moreno-Franco P Pickering B Herasevich V. A multisite survey study of EMR review habits, information needs, and display preferences among medical ICU clinicians evaluating new patients. Appl Clin Inform. (2017) 8:1197–207. doi: 10.4338/ACI-2017-04-RA-0060, PMID: 29272901 PMC5802307

[40] Ahmed A Chandra S Herasevich V Gajic O Pickering BW. The effect of two different electronic health record user interfaces on intensive care provider task load, errors of cognition, and performance. Crit Care Med. (2011) 39:1626–34. doi: 10.1097/CCM.0b013e31821858a0, PMID: 21478739

[41] Herasevich S Pinevich Y Lipatov K Barwise AK Lindroth HL LeMahieu AM. Evaluation of digital health strategy to support clinician-led critically ill patient population management: a randomized crossover study. Crit Care Explor. (2023) 5:e0909. doi: 10.1097/CCE.0000000000000909, PMID: 37151891 PMC10158897

[42] Favre-Félix J Dziadzko M Bauer C Duclos A Lehot JJ Rimmelé T. High-Fidelity simulation to assess task load index and performance: a prospective observational study. Turk J Anaesthesiol Reanim. (2022) 50:282–7. doi: 10.5152/TJAR.2022.21234, PMID: 35979975 PMC9524413

[43] Wang F Kaushal R Khullar D. Should health care demand interpretable artificial intelligence or accept "black box" medicine? Ann Intern Med. (2020) 172:59–60. doi: 10.7326/M19-2548 31842204

[44] Armeni P Polat I De Rossi LM Diaferia L Meregalli S Gatti A. Digital twins in healthcare: is it the beginning of a new era of evidence-based medicine? A critical review. J Pers Med. (2022) 12:1255. doi: 10.3390/jpm12081255 36013204 PMC9410074

[45] Kühne F Schomaker M Stojkov I Jahn B Conrads-Frank A Siebert S. Causal evidence in health decision making: methodological approaches of causal inference and health decision science. Ger Med Sci. (2022) 20:Doc12. doi: 10.3205/000314 36742460 PMC9869404

[46] Bray A Webb JB Enquobahrie A Vicory J Heneghan J Hubal R. Pulse physiology engine: an open-source software platform for computational modeling of human medical simulation. SN Compr Clin Med. (2019) 1:362–77. doi: 10.1007/s42399-019-00053-w

[47] Dang J Lal A Flurin L James A Gajic O Rabinstein AA. Predictive modeling in neurocritical care using causal artificial intelligence. World J Crit Care Med. (2021) 10:112–9. doi: 10.5492/wjccm.v10.i4.112, PMID: 34316446 PMC8291004

[48] Lal A Pinevich Y Gajic O Herasevich V Pickering B. Artificial intelligence and computer simulation models in critical illness. World J Crit Care Med. (2020) 9:13–9. doi: 10.5492/wjccm.v9.i2.13, PMID: 32577412 PMC7298588

[49] Linkov I Galaitsi S Trump BD Keisler JM Kott A. Cybertrust: from explainable to actionable and interpretable artificial intelligence. Computer. (2020) 53:91–6. doi: 10.1109/MC.2020.2993623

[50] Lal A Dang J Nabzdyk C Gajic O Herasevich V. Regulatory oversight and ethical concerns surrounding software as medical device (SaMD) and digital twin technology in healthcare. Ann Transl Med. (2022) 10:950. doi: 10.21037/atm-22-4203 36267783 PMC9577733

