# Supplementary material for: Development and usability testing of a patient digital twin for critical care education: a mixed methods study
Source: Front Med (Lausanne). 2024 Jan 11;10:1336897. doi: 10.3389/fmed.2023.1336897 (PMC10808677; doi:10.3389/fmed.2023.1336897)

Supplementary Material

# Supplementary Data

## Digital twin patient model design and coding

Actionable variables for each organ system, named "organ system variables," are clinical markers that clinicians can objectively assess and modify using therapeutic interventions and that are displayed in the ICU EHR. Examples include patient vital signs, physical examination findings, and diagnostic test results (laboratory and radiologic). Numerical variables are classified into different levels based on their value, where 0 corresponds to the normal variable range and -1, -2, 1, and 2 correspond to value ranges that are lower or higher than normal. Categorical variables are classified as absent (0) or present (1). Each organ system variable value is also color coded as "white," "yellow," and "red" based on its degree of disturbance from normal. White color indicates that a clinical variable is in its normal range and no intervention is needed, while yellow or red colors indicate a variable disturbance that would typically require action. Medications and other clinical interventions, such as transfusions, are coded in two levels based on their dose.

Expert rules describe the interaction between different organ system variables and the effects of various clinical interventions. Each rule is comprised of:

- Input: the change in a clinical marker or the administration of a clinical intervention that triggers the onset of the rule.
- Output: the organ system variable that is affected by the rule. Each input can influence one or more output variables.
- Effect: a description of the direction (increase or decrease) of the rule’s effect.
- Onset: the time required before the change in the output variable will occur.
- Duration: the time required before the output variable will return to its previous value.
- Probability: the likelihood that the rule will be triggered.

Two examples of expert rules are presented in **Supplementary Table 1**. The first rule describes an interaction between two organ system variables and states that if the mean arterial pressure (MAP) is < 65 mmHg for 60 minutes, then in 80% of the cases, the lactate level will increase. This rule lasts until the input is satisfied, meaning lactate will decrease to its previous level only when MAP returns ≥ 65 mmHg. The second rule describes the effect of norepinephrine infusion on MAP. It states that when a norepinephrine infusion is started, 2 minutes later, MAP will increase, and this increase in MAP will last until the norepinephrine infusion is turned off for at least 2 minutes. In a simulated scenario, a hypotensive patient will have high lactate levels, and if norepinephrine infusion is ordered, MAP will increase, and lactate will clear. The effect size, i.e., how much MAP or lactate will increase, depends on the input level, so a higher dose of norepinephrine will trigger a higher MAP increase, and a lower MAP value will lead to a higher lactate value.

# Supplementary Tables and Figures

**Supplementary Table 1.** Expert rules examples

| **Rule** | **Input** | **Output** | **Effect** | **Onset** | **Duration** | **Probability** |
| --- | --- | --- | --- | --- | --- | --- |
| IF MAP < 65 mmHg, THEN lactate increases | MAP < 65 mmHg | Lactate | Increase | 60 min | Until MAP ≥ 65 mmHg | 80% |
| Norepinephrine IV infusion increases MAP | Norepinephrine IV infusion | MAP | Increase | 2 min | 2 min after the infusion is stopped | 100% |

Abbreviations: MAP, mean arterial pressure; IV, intravenous; min, minutes

**Supplementary Figure 1.** Physiologic interactions between the organ system variables described by expert rules


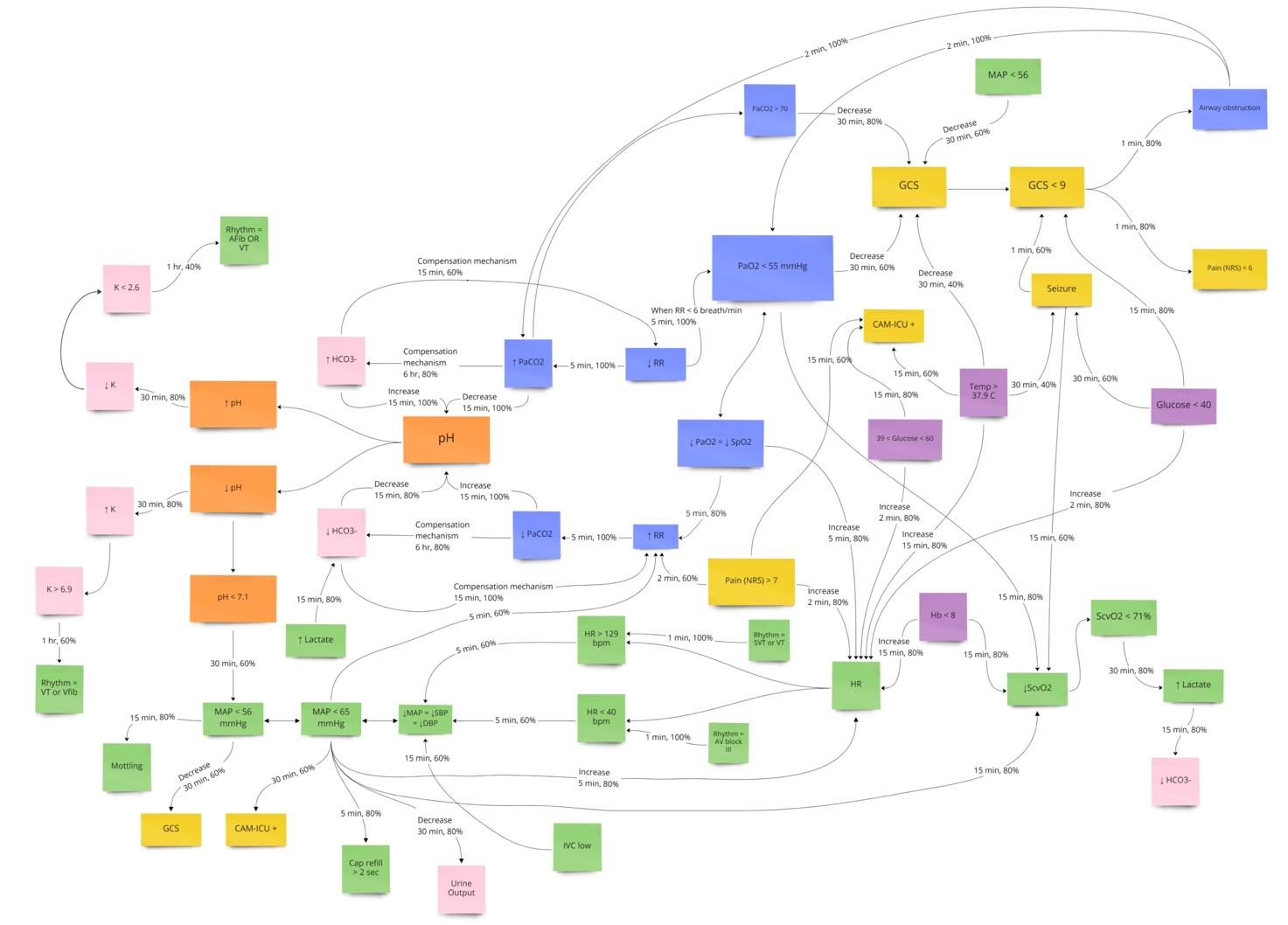

Supplement: Supplementary file 1 [file Data_Sheet_1.DOCX]
